# Adherence to Long-Acting Injectable Risperidone ISM in a Community Psychiatric Setting: A Real-World Observational Study

**DOI:** 10.1192/j.eurpsy.2026.12143

**Published:** 2026-07-31

**Authors:** G. Cereda, A. Panariello, A. Galluzzo, D. Bortoletti, S. Giaretto, A. Vita, M. Percudani

**Affiliations:** 1 Department of Mental Health and Addiction Services, Niguarda Hospital, Milan; 2 Department of Clinical and Experimental Sciences, University of Brescia, Brescia, Italy

## Abstract

**Introduction:**

Schizophrenia affects over 24 million people worldwide and is a leading cause of disability (Collaborators Eur Neuropsychopharmacol 2024; 93: 1-12).

Antipsychotics are the cornerstone of treatment, reducing symptoms and relapse (Leucht et al. Lancet 2012; 379: 2063-2074). Yet up to 50% of patients are non-adherent (Llorca et al. Eur Psychiatry 2013; 28: 1-8). As many as 60% discontinue after 3 months and 80% after 2 years, with a five-fold higher relapse risk in those who stop treatment (Leijala et al. BMC Psychiatry 2021; 21: 37). Long-acting injectables (LAIs) were developed to improve adherence and detect missed doses (Correll et al. CNS Drugs 2021; 35: 215-233). Risperidone ISM, a once-monthly LAI without oral supplementation, has proven efficacy in trials, but real-world data are scarce. Meta-analyses confirm LAIs reduce relapse and hospitalization compared with oral antipsychotics (Kishimoto et al. Lancet Psychiatry 2021; 8: 387-404).

**Objectives:**

To assess adherence to risperidone ISM in a community psychiatric setting, comparing adherent and non-adherent patients, and evaluating rehospitalizations and outpatient visits.

**Methods:**

This retrospective study included 44 patients who initiated risperidone ISM, all diagnosed with a psychotic spectrum disorder. Patients with fewer than three injections (n=10) or dropout within the first three injections (n=3) were excluded. The final sample comprised 31 patients. Adherence was defined as continuation without dropout (n=21). Non-adherence was defined as dropout after ≥4 injections (n=10). Demographic and clinical data were collected. Outcomes were rehospitalizations and outpatient visits, standardized by months of follow-up. Chi-square and Mann–Whitney U tests were used.

**Results:**

No significant differences were found in age, sex, or substance use. Schizophrenia was more frequent among adherent patients compared with other psychotic spectrum disorders. Clinical outcomes are shown in Table 1: mean rehospitalization rates were low in both groups (0.022 vs 0.050 per month, p=0.56). Outpatient visits were more frequent among adherents (2.37 vs 2.07 per month), though not significant (p=0.11).

**Table 1.** Demographic and clinical characteristics of the sample Table 1. Long description.

Variable	Adherent	Non-adherent	p-value
Age, mean ± SD	42.1 ± 11.3	40.7 ± 10.8	0.72
Sex, male %	61.9	60.0	0.91
Schizophrenia diagnosis %	66.7	40.0	0.04*
Rehospitalizations/month	0.022 ± 0.08	0.050 ± 0.11	0.56
Outpatient visits/month	2.37 ± 0.83	2.07 ± 1.77	0.11

**Conclusions:**

Two-thirds of patients remained adherent to risperidone ISM. Adherence was higher in schizophrenia than in other psychotic spectrum disorders. While rehospitalizations did not differ, adherent patients tended to maintain greater outpatient engagement. These findings stress the importance of continuity of care in sustaining LAI adherence.

**Disclosure of Interest:**

None Declared

## Long descriptions

### Table 1. Long description

The table consists of four columns: Variable, Adherent, Non-adherent, and p-value.

* Age, mean plus or minus S D: Adherent 42.1 plus or minus 11.3, Non-adherent 40.7 plus or minus 10.8, p-value 0.72.

* Sex, male percentage: Adherent 61.9, Non-adherent 60.0, p-value 0.91.

* Schizophrenia diagnosis percentage: Adherent 66.7, Non-adherent 40.0, p-value 0.04 asterisk.

* Rehospitalizations per month: Adherent 0.022 plus or minus 0.08, Non-adherent 0.050 plus or minus 0.11, p-value 0.56.

* Outpatient visits per month: Adherent 2.37 plus or minus 0.83, Non-adherent 2.07 plus or minus 1.77, p-value 0.11.

Navigate back to Table 1..

